# High-Sensitivity C-Reactive Protein is a Predictor of Subsequent Atrial High-Rate Episodes in Patients with Pacemakers and Preserved Ejection Fraction

**DOI:** 10.3390/jcm9113677

**Published:** 2020-11-16

**Authors:** Min-Tsun Liao, Chun-Kai Chen, Ting-Tse Lin, Li-Ying Cheng, Hung-Wen Ting, Yen-Bin Liu

**Affiliations:** 1 Division of Cardiology, Department of Medicine, National Taiwan University Hospital Hsinchu Branch, Hsinchu 300, Taiwan; liaomintsun@gmail.com (M.-T.L.); ineosky@gmail.com (C.-K.C.); ttlin111@gmail.com (T.-T.L.); chengliying2@gmail.com (L.-Y.C.); sharon3458@gmail.com (H.-W.T.); 2 Institute of Epidemiology and Preventive Medicine, College of Public Health, National Taiwan University, Taipei 100, Taiwan; 3 College of Medicine, National Taiwan University, Taipei 100, Taiwan; 4 Division of Cardiology, Department of Medicine, National Taiwan University Hospital, Taipei 100, Taiwan

**Keywords:** atrial fibrillation, pacemaker, atrial high-rate episodes, hs-CRP, inflammatory markers

## Abstract

Atrial fibrillation (AF) is associated with morbidity and mortality. Modern pacemakers can detect atrial high-rate episodes (AHREs) as a surrogate for AF. It remains controversial whether inflammation is a cause or a consequence of AF. This study investigated whether the inflammatory biomarker high-sensitivity C-reactive protein (hs-CRP) can predict subsequent AHREs. This study gathered prospective data from patients with pacemakers and a left ventricle EF ≥ 50% between 2015 and 2019. The hs-CRP and other cardiac biomarkers at baseline and device-detected AHREs, defined as atrial rate ≥ 180 bpm and duration ≥ 6 min, were determined. Cox regression analysis was used to estimate the independent predictors for AHREs. A total of 171 consecutive patients were included. During the median follow-up of 614 days, 66 patients (39%) developed subsequent AHREs. In the univariate Cox regression analysis, sick sinus syndrome (*p* = 0.005), prior AF (*p* < 0.001), mitral A velocity (*p* = 0.008), and hs-CRP (*p* = 0.013) showed significant association with the increased risk of AHREs. In the multivariate Cox regression model, hs-CRP (HR = 1.121, 95% confidence interval = 1.015–1.238, *p* = 0.024) retained its significance. Our results suggest that elevated hs-CRP could predict subsequent AHREs and that inflammation could play a role in AF pathogenesis in patients with preserved EF.

## 1. Introduction

Atrial fibrillation (AF) is the most common arrhythmia and is associated with various morbidities and mortality [1,2]. AF is also responsible for a significant number of hospitalization and mortality events in heart failure (HF) with reduced ejection fraction (HFrEF) or preserved ejection fraction (HFpEF) patients [3]. AF is more prevalent and independently associated with poor clinical outcomes in patients with HFpEF than patients with HFrEF are [4,5]. Thus, AF may have a different pathogenesis in patients with reduced EF and preserved EF. The causes of AF are multi-factorial, and the pathogenesis of AF is incompletely understood. Substantial evidence has linked inflammation to the initiation and perpetuation of AF. However, it remains elusive whether inflammation is a consequence or a cause of AF [6]. Moreover, it is unclear whether inflammation has the same effect in normal atria and in atria with substantial structural remodeling [7].

Modern pacemakers provide a continuous monitoring of heart rhythm, which can detect atrial high-rate episodes (AHREs) [8,9]. Previous studies have shown that AHREs are associated with an increased risk of new-onset AF, thromboembolism, heart failure, and cardiovascular mortality [10,11,12,13]. The current guidelines recommend that if AHREs, defined as atrial rate ≥ 180 beats per minute and duration ≥ 5–6 min, are detected, further examinations are suggested to diagnose AF for determining adequate treatments [14]. AF could progress from asymptomatic AF to paroxysmal AF, persistent AF, and permanent AF [15]. Significant benefits could be obtained with interventions at an early stage of AF. New-onset AHREs could be used as a surrogate of AF at its early stage. The purpose of this study was to investigate whether inflammatory biomarkers (high-sensitivity C-reactive protein, hs-CRP) and/or other cardiac biomarkers, including makers of myocardial damage (high-sensitivity cardiac troponin T, hs-cTnT), myocyte stretch (N-terminal pro-brain natriuretic peptide, NT-proBNP), and coagulation (D-dimer), can predict the future occurrence of AHREs ≥ 6 min in patients with pacemakers and preserved EF.

## 2. Experimental Section

### 2.1. Study Design and Population

This study was a prospective analysis of data from patients with cardiovascular electronic devices and left ventricle ejection fraction (LVEF) ≥ 50% between 2015 and 2019 in our single-center hospital. The inclusion criteria were (1) patients with pacemakers due to sick sinus syndrome (SSS) or high-degree atrioventricular block, (2) age 20 to 100 years, and (3) pacemakers implanted for ≥30 days with no complications. The exclusion criteria were (1) permanent and long-standing persistent AF, (2) pacemakers with only a single lead or mode that cannot record AHREs, (3) implantable cardioverter defibrillators and cardiac resynchronization therapy, (4) lack of informed consent, (5) unwillingness or inability to return for follow-up visits or reasons to believe that adherence to follow-up visits would be irregular, (6) current or scheduled enrollment in other conflicting studies, and (7) concomitant disease or other medical conditions that are likely to result in death within 6 months. At the beginning of patient enrollment, medical histories, including demographics and medication, were carefully recorded. Echocardiography and blood sampling to test the levels of NT-pro-BNP, hs-CRP, hs-cTnT, and D-dimer were performed. This study was approved by the Institutional Review Board of National Taiwan University Hospital Hsin-Chu Branch, and all the participants provided written informed consent.

### 2.2. Echocardiography

An echocardiographic ultrasonic system (IE33, Philips; Andover, MA, USA) with an S5 transducer was used for the evaluations of cardiac function and shape. Two-dimensional, M-mode, and Doppler ultrasound recordings were used to measure the dimensions of the left ventricular, ventricular septum, posterior wall, and left atrial diameter. LVEF by M-mode was measured via the parasternal long-axis view, and the diastolic function of the left ventricle was evaluated by mitral flow and early (E) and late (A) ventricular filling velocities according to the American Society of Echocardiography guidelines [16]. Left ventricular mass index (LVMI) was calculated by a method described in Devereux et al. [17], while LV end-diastolic and end-systolic volumes were calculated by the Teichholz method [18]. The standardized measurements of inter- and intra-observer variability were investigated in our echocardiography lab, and the inter- and intra-observer variabilities of the mean mitral E’ were 2.38% and 1.67%, respectively [19].

### 2.3. Cardiac Biomarkers

All laboratory examinations were performed according to our standardized procedures. We additionally checked the levels of NT-proBNP, hs-CRP, hs-cTnT, and D-dimer as representative of heart failure markers, inflammation markers, cardiac damage markers, and thrombosis markers, respectively. This procedure was performed primarily to explore the early mechanisms that can lead to AF development. The hs-CRP concentration was obtained from serum samples (AU680 Clinical Chemistry Analyzer, Beckman Coulter, Kowloon, Hongkong). The NT-pro-BNP and hs-cTnT concentrations were obtained from plasma samples (Roche Cobas E411, Roche Diagnostics, Kowloon Bay, Hongkong). The D-dimer concentration was obtained from plasma samples (Automated Blood Coagulation Analyzer CS-1600, Sysmex, Kowloon, Hong Kong). The hs-CRP level was determined with the latex particle immunoturbidimetric method. The NT-pro-BNP and hs-cTnT concentrations were measured using the electrochemiluminescence immunoassay (ECLIA) method. The D-dimer concentration was measured using the Immun-Nephelometry method. The normal concentrations of NT-pro-BNP, hs-CRP, hs-cTnT, and D-dimer are 0.0~124.9//0.0~449.9 pg/mL (<75 years and ≥75 years), <1.0 mg/dL, <14 ng/L, and 0~0.549 mg/L, respectively.

### 2.4. AHREs Endpoint and Clinical Follow-Up

We evaluated whether cardiac biomarkers can predict the occurrence of AHREs. Patients were primarily followed up in the outpatient department every 3 or 6 months, and the function of the pacemakers was also checked. Data from each visit were collected as part of the electronic medical records, which contain the time and duration of each AHRE and various other features. The pacemakers recorded subsequent AHREs, along with the time of occurrence, the duration of AHREs, and the maximal atrial rate. All the records were reviewed independently by two cardiac electrophysiologists (MTL, TTL). The AHREs endpoint was determined according to the recommendations of the ESC guidelines [14] and the results of previous research [10]. The AHREs endpoint was defined as an atrial rate ≥ 180 bpm and duration ≥ 6 min. Accordingly, the levels of cardiac biomarkers at baseline and at the time of device-detected AHRE episodes ≥ 6 min were determined. The AHREs burden was defined as the average percentage of AHRE duration per day through all the follow-up time in each patent.

### 2.5. Statistical Analysis

All the data were represented as mean ± standard deviation (SD) and numbers (percentage). Continuous variables were compared by the Student’s t-test for normally distributed data and the Mann–Whitney U test for non-normally distributed data. Categorical variables were compared by means of the Chi-square or Fisher, exact test. The baseline variables, including basic characteristics, medications, parameters of cardiac echography, and cardiac biomarkers, were evaluated by a Cox univariate regression analysis to evaluate subsequent AHREs and by the univariate linear regression analysis to evaluate the AHRE burden in patients with subsequent AHREs. Additionally, age, gender, and any variable presenting a *p* value below 0.1 were studied in the Cox multivariate regression analysis and the multivariate linear regression analysis using a stepwise forward method. A receiver operating characteristic (ROC) curve was used to determine the optimal cut-point value of cardiac biomarkers, which is defined as the value whose sensitivity and specificity are closest to the value of the area under the ROC curve. A logistic regression-based prediction model for binary outcome was used to predict subsequent AHREs. Since there was a different follow-up time, we used the integrated discrimination index (IDI) and net reclassification index (NRI) to analyze the individual time-dependent estimated risk of AHREs. The cumulative proportional probabilities of becoming free from AHREs were analyzed by Kaplan–Meier curves and the Log Rank test. To further investigate the potential link between hs-CRP and incident AHRE, we performed a sensitivity analysis to evaluate non-prior AF patients by the Cox univariate and multivariate regression analysis. A subdistribution hazard model was also used to evaluate the competing risk of mortality. Statistical analysis was performed using SPSS version 22.0 for Windows (SPSS Inc., Chicago, IL, USA) and R Statistics version 3.6.2 for Windows (The R Foundation for Statistical Computing, Vienna, Austria). A *p* value < 0.05 was considered statistically significant.

## 3. Results

### 3.1. Study Population and Echocardiography

A total of 171 consecutive patients were evaluated; the mean age was 74.1 ± 11.5 years and 48.5% of the patients were women. During the median (interquartile range) follow-up of 614 (916) days, 66 patients developed subsequent AHREs ≥ 6 min. The patients who had subsequent AHREs had a significantly greater incidence of SSS and prior AF (defined as except permanent and long-standing persistent AF) than those patients without AHREs. The number of deaths was 16 during follow-up, with 8 in the AHRE group and 8 in the non-AHRE group. The age, gender, body weight, and risk factors of stroke and medication intake were comparable between the two groups. A trend of a higher level of hs-CRP was observed in patients with AHREs than in patients without AHREs. In the echocardiography, the patients with AHREs had a significantly lower mitral A velocity than those patients without AHREs. Other echocardiographic parameters, such as chamber size, thickness, LVEF, LVM, LVMI, left atrial dimension, and mitral E velocity, were comparable between the two groups. These data are shown in Table 1.

### 3.2. Univariate and Multivariate Predictors of Subsequent AHREs

In the univariate Cox regression analysis, SSS (*p* = 0.005), prior AF (*p* < 0.001), mitral A velocity (*p* = 0.008), and hs-CRP (*p* = 0.013) showed significant associations with the increased risk of AHREs ≥ 6 min. Age, gender, SSS, prior AF, betablocker, mitral A velocity, NT-pro-BNP, and hs-CRP were adjusted in the multivariate Cox regression analysis. When the biomarkers were tested in the multivariate Cox regression model, hs-CRP (HR = 1.121, 95% confidence interval (CI) = 1.015–1.238, *p* = 0.024) retained its significance in predicting AHREs. SSS, prior AF, and mitral A velocity were also significant in predicting AHREs (HR = 1.900, 95% CI = 1.083–3.333, *p* = 0.025; HR = 2.797, 95% CI = 1.452–5.388, *p* = 0.002; HR = 0.989, 95% CI = 0.979–0.999, *p* = 0.038, respectively). These results are shown in Table 2.

### 3.3. Univariate and Multivariate Analysis for AHRE Burden

In the univariate linear regression analysis of patients with subsequent AHREs, prior AF (*p* = 0.008), CHF (*p* = 0.001), hypertension (*p* = 0.050), and hs-CRP (*p* = 0.037) showed significant associations with AHREs burden. Age, gender, prior AF, CHF, hypertension, COPD, and hs-CRP were adjusted in the multivariate linear regression analysis. When the biomarkers were tested in the multivariate linear regression model, hs-CRP (standardized beta = 0.223, *p* = 0.038) retained its significance in association with the AHRE burden. Prior AF and CHF were also significant in association with the AHRE burden (standardized beta = 0.279, *p* = 0.013; standardized beta = 0.321, *p* = 0.004, respectively). These results are shown in Table 3.

### 3.4. Hs-CRP and the Risk of AHREs

**Figure 1:**
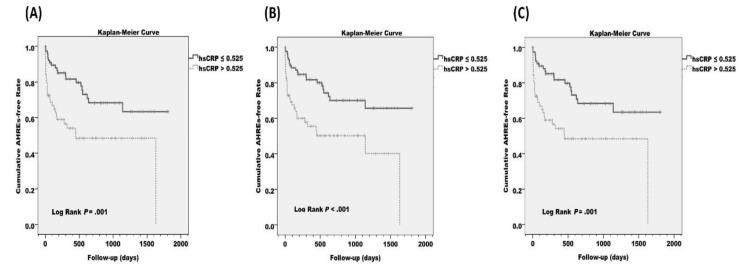
Estimated cumulative atrial high-rate episode (AHRE)-free survival (Kaplan–Meier) analysis between high-sensitivity C-reactive protein (hs-CRP) > 0.525 mg/L and hs-CRP ≤ 0.525 mg/L in (**A**) all patients, (**B**) patients without prior atrial fibrillation (AF), and (**C**) patients without prior AF and congestive heart failure (CHF). The cut-point value was optimized from the receiver operating characteristics (ROC) curves.

The optimized cut-point value of hs-CRP, where the sensitivity (0.379) and specificity (0.933) are closest to the value of the area under the ROC curve (0.699), was 0.525 mg/L. These results are shown in Supplementary Figure S1. The estimated cumulative AHRE-free survival (Kaplan–Meier) analysis demonstrated a significant difference between hs-CRP > 0.525 mg/L and hs-CRP ≤ 0.525 mg/L (log rank *p* = 0.001). Such a difference was also observed in the Kaplan–Meier analysis in patients without prior AF and without prior AF and congestive heart failure (CHF) (log rank *p* < 0.001 and *p* = 0.001, respectively). These results are shown in Figure 1.

### 3.5. Prediction Model, Discrimination, and Reclassification

**Figure 2:**
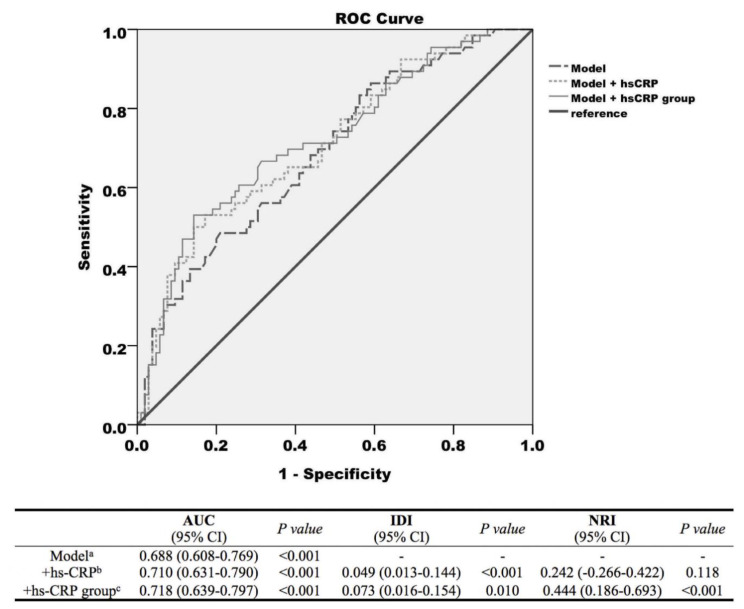
Receiver operating characteristic (ROC) curves from logistic regression models predicting subsequent atrial high-rate episodes (AHREs) ≥ 6 min. (1) Model (long dotted line) with the variables sick sinus syndrome (SSS), prior atrial fibrillation (AF), and mitral valve (MV) A wave. (2) Model + high-sensitivity C-reactive protein (hs-CRP) level (short dotted line). (3) Model + hs-CRP group (cut-point value 0.525 mg/L, solid line) and discrimination and reclassification accuracy of the risk markers of atrial high-rate episodes. ^a^ The model included the clinical parameters, which remained significant predictors of AHREs in the Cox multivariate hazards model; ^b^ hs-CRP as a continuous variable was added to the model; ^c^ hs-CRP as a dichotomized variable was added to the model. The optimized cut-point value of hs-CRP obtained by receiver operating characteristics was 0.525 mg/L. Abbreviations: AUC, area under the curve; IDI, Integrated discrimination index; NRI, net reclassification index; hs-CRP, high-sensitivity C-reactive protein.

ROC curves from logistic regression models predicting subsequent AHREs ≥ 6 min and the discrimination and reclassification upon the addition of hs-CRP to the established model for the prediction of subsequent of AF are presented in Figure 2. The prediction model of subsequent AHREs ≥ 6 min included the clinical variables SSS, prior AF, and MV. A wave showed that the area under the curve (AUC) was 0.688 (95% CI = 0.608–0.769, *p* < 0.001). With the inclusion of hs-CRP as a continuous variable in the established model, the AUC was 0.710 (95% CI = 0.631–0.790, *p* < 0.001), and significant improvements were observed in IDI (*p* < 0.001) but not NRI (*p* = 0.118). With the inclusion of hs-CRP as a dichotomized variable (cut–point value 0.525 mg/L) in the established model, the AUC was 0.718 (95% CI = 0.639–0.797, *p* < 0.001) and significant improvement was observed in both IDI and NRI (*p* = 0.010 and *p* < 0.001, respectively). However, the AUCs of the prediction model, even with the addition of hs-CRP as a dichotomized variable, remained relatively modest in the prediction of subsequent AHREs ≥ 6 min.

### 3.6. Sensitivity Analysis

In the univariate Cox regression analysis of non-prior AF patients, SSS (*p* = 0.046), mitral A velocity (*p* = 0.015) and hs-CRP (*p* = 0.014) showed significant associations with the increased risk of AHREs ≥ 6 min. Age, gender, SSS, beta-blockers, mitral A velocity, and hs-CRP were adjusted in the multivariate Cox regression analysis. Hs-CRP (HR = 1.155, 95% CI = 1.038–1.285, *p* = 0.008) retained its significance in predicting AHREs. These results are shown in Supplementary Table S1.

In the univariate subdistribution hazard model, SSS (*p* = 0.002), prior AF (*p* < 0.001), mitral A velocity (*p* = 0.009), and hs-CRP (*p* = 0.014) showed significant associations with the increased risk of AHREs ≥ 6 min. Age, gender, SSS, prior AF, mitral A velocity, NT-pro-BNP, and hs-CRP were adjusted in the multivariate Cox regression analysis. Hs-CRP (HR = 1.122, 95% CI = 1.014–1.242, *p* = 0.026) retained its significance in predicting AHREs. These results are shown in Supplementary Table S2.

## 4. Discussion

The main findings of this prospective study with a median follow-up of nearly 2 years are (1) nearly 40% of patients with pacemakers have device-detected AHREs ≥ 6 min during the follow-up period; (2) the prior AF, SSS, mitral A velocity, and hs-CRP value independently correlated with subsequent AHREs; (3) after adjusting the relevant predictors of subsequent AHREs, the hs-CRP level retained its significance in predicting the occurrence of subsequent AHREs; (4) the hs-CRP level was also significantly associated with the AHRE burden; (5) the optimized cut-point value > 0.525 mg/L of hs-CRP had a significant discrimination and reclassification accuracy in predicting subsequent AHREs; and (6) only the baseline inflammatory biomarker hs-CRP, but not other cardiac biomarkers (hs-cTnT, NT-proBNP, or D-dimer), was significantly associated with the subsequent AHREs. Thus, our results imply that inflammation could be involved in the pathogenesis of AF at an early stage without prominent cardiac structural remodeling and clinically evident heart failure. If inflammation is involved in the underlying mechanisms of AF in patients with preserved EF, modification of the inflammatory substrates could be the target of therapeutic interventions to terminate or prevent AF.

### 4.1. Hs-CRP as a Predictor of AHREs

The hs-CRP is a marker of inflammation, and the FOURIER study (Further Cardiovascular Outcomes Research with PCSK9 Inhibitor in Patients with Elevated Risk) showed that the hs-CRP level can predict the risk of various cardiovascular diseases, including cardiovascular death, non-fatal myocardial infarction, non-fatal stroke, hospitalization for acute coronary syndrome, and coronary revascularization [20]. Previous studies have revealed that inflammation biomarkers are good predictors of the occurrence and recurrence of AF. Aviles et al. performed a cross-sectional study of 5806 subjects and reported that the baseline levels of CRP were higher in patients affected by AF even after adjustment for multiple variables potentially associated with AF, and that the CRP level is a strong predictor of future AF [21]. Marott et al. monitored 10,276 subjects for 12 to 15 years for the incidence of AF and showed that the CRP levels in the upper vs. lower quintiles were associated with a 2.19-fold increase in the risk of AF after adjustment for age, sex, and statin usage [22]. In a population-based cohort of 1011 patients, patients with high CRP and high complement levels had a significantly higher risk of AF than those with normal CRP and low complement levels; however, the absence of a high CRP level was not significantly associated with AF [23]. Additionally, a high level of CRP determined prior to cardioversion acted as an independent predictor of AF recurrence after cardioversion and the maintenance of sinus rhythm after cardioversion resulted in a gradual decrease in the CRP level [24]. Other investigators found a correlation between the baseline CRP levels and the risk of recurrent AF after catheter ablation [25]. While Nortamo et al. reported that hs-CRP ≥ 1.8 mg/L was associated with the new occurrence of clinical AF [26], our study showed that hs-CRP > 0.525 mg/L was associated with subsequent AHREs ≥ 6 min. The lower cut–point value of hs-CRP in our study could have resulted from using AHREs, and not AF, as an endpoint.

In our multivariate Cox regression analysis, prior AF were also significant in predicting AHREs. A sensitivity analysis in non-AF patients further clarify the link between hs-CRP and AHREs. The mean age of our study papulation is nearly 74 years and the total mortality rate was about 9.4% during follow-up. The mortality may have a competitive relationship with AHRE incidence. The estimation of the censoring distribution can affect the accuracy and conclusions of a competing risks analysis, so it is important that mortality should be considered when analyzing time-to-event data in the presence of competing risks. Our finding is robust, with sensitivity analyses indicating that the predictability of hs-CRP for AHREs is preserved in a proportional subdistribution hazards model for mortality.

### 4.2. NT-proBNP, hs-cTnT, and D-dimer and the Risk of AHREs

Hijazi et al. reported that the levels of NT-proBNP, hs-cTnT, and D-dimer can significantly predict the risk of stroke/systemic embolic events, major bleeding, and mortality within 12 months following a diagnosis of AF [27]. The ARIC (Atherosclerosis Risk in Communities) study analyzed 9556 patients without HF and found that the NT-proBNP concentration had a significant correlation with future AF [28]. Nakanishi et al. reported that the level of hs-cTnT can be significantly used to predict the probability of recurrence within one year of AF patients receiving catheter ablation [29]. Therefore, the levels of NT-proBNP and hs-cTnT seem to be significant predictors of subsequent or recurrent AF. To the best of our knowledge, previous studies have not explored whether the NT-pro-BNP or hs-cTnT levels can be used to predict the risk of subsequent AHREs in patients with pacemakers and preserved EF. Our study found that NT-proBNP and hs-cTnT could not be used to predict the possibility of subsequent AHREs.

The risk of stroke in patients with AHREs is lower than that in patients with AF [30], and there is no obvious temporal relationship between AHREs and stroke [31]. This may imply that the mechanism of stroke in patients with AHREs may differ from that in patients with AF. Our study revealed that there was no significant association between D-dimer concentration and subsequent AHREs. As a result, the biomarkers of coagulation and D-dimer concentration did not increase significantly in patients with AHREs and preserved EF.

### 4.3. AHREs Risk Prediction Model

Most of the previous studies have evaluated the future occurrence of AF, and only a few studies have analyzed the factors that could predict the occurrence of AHREs. In the AHRE risk prediction model, Pastori et al. reported that age, prior AF, white cell count, and CRP were independent predictors of AHRE incidents [32]. The hazard ratio of high CRP level (above median) for subsequent AHREs was 1.039 in the median follow-up of 16.5 months. However, the mean EF was 46.5 ± 18.0%, and 39.6% of the patient cohort had a history of CHF. In general, our findings are consistent with those of prior studies. Our study population was more homogeneous and the patients did not have structural heart diseases, since all of them had an EF ≥ 50% and only 11.7% of them had a history of CHF. We found that the hs-CRP level was as good a predictor as the CRP level in patients with preserved EF. Furthermore, the predictive power of the hs-CRP level was even more significant in our patients with preserved EF. With the inclusion of hs-CRP as a dichotomized variable (cut-point value 0.525 mg/L) in the established model, the AUC was 0.718 (*p* < 0.001) and significant improvement was observed in both IDI and NRI.

In addition, our research found that the diagnosis of SSS and low mitral A velocity are also independent predictors of AHREs. It should be noted that some SSS patients could potentially have undiagnosed AF [33]. Thus, SSS can be used as a risk factor for the occurrence of AHREs in patients with a preserved EF. The mitral A velocity represents the peak velocity flow in late diastole during left ventricle relaxation caused by atrial contraction. Low mitral A velocity, suggesting the adverse functional remodeling of the left atrium, is also associated with subsequent AHREs in patients with preserved EF. Additionally, P wave and intra–atrial block have previously been reported to be predictors of subsequent AHREs [34].

### 4.4. Inflammation and AF Pathogenesis in Patients with Preserved EF

The pathogenesis of AF is incompletely understood. The current understanding of a correlation between inflammation and AF could be summarized in the following three aspects: (1) inflammation has a role in AF pathogenesis, (2) AF generates an inflammatory response, and (3) elevation in CRP levels during AF is a consequence of heart failure [6].

The role of atrial inflammation in the pathogenesis of AF has also not been fully elucidated, although histologic evidence of inflammation was reported in 66% of atrial biopsy specimens from patients with lone AF. Thus, it remains controversial as to whether inflammation is a consequence or a cause of AF [35]. The continuous monitoring of atrial rhythm by a pacemaker has provided a unique platform to better understand longitudinal physiological alterations in inflammation and AF. In our study, the baseline inflammatory biomarker hs-CRP, but not other cardiac biomarkers (hs-cTnT, NT-proBNP or D-dimer), was an independent predictor of subsequent AHREs in patients with pacemakers and preserved EF. Even after excluding patients with a history of prior AF and/or HF, the hs-CRP level retained its significance in predicting AHREs. Meanwhile, the hs-CRP level was also significantly associated with the AHRE burden. Our results imply that inflammation could be involved in the pathogenesis of AF at an early stage without prominent cardiac structural remodeling and clinically evident heart failure.

### 4.5. Study Limitation

This study has some limitations. First, this is a single-center study. The number of patients was not large and the findings may not be extrapolated to all patients with pacemakers. However, our research showed that approximately 40% of patients with pacemakers have device-detected AHREs ≥ 6 min during a 2-year follow-up, and these data are in agreement with previously published ASSERT (Asymptomatic atrial fibrillation and Stroke Evaluation in pacemaker patients and the AF reduction atrial pacing Trial) data that showed that 34.7% of patients had AHREs ≥ 6 min during a mean follow-up of 2.5 years [8]. Our study was reasonable in finding factors that were able to predict the occurrence of subsequent AHREs. Second, in our study, the results of the prediction model showed a modest discrimination. Therefore, the impacts of other confounding factors cannot be overruled. These include chronic inflammation caused by various other diseases, such as gastroesophageal reflux or autoimmune disease. Third, this study used baseline biomarkers to predict the possibility of the subsequent occurrence of AHREs without any follow-up biomarkers. As such, doubt remains as to whether baseline biomarkers are effective in predicting the occurrence of AHREs. Finally, AHREs do not accurately represent AF and could result in a false-positive representation of AF due to noise, repetitive non-re-entrant ventriculoatrial synchrony, and far-field R-wave over-sensing [8]. Moreover, this study did not record all the electrograms of AHREs. Thus, our study cannot discriminate whether AHREs accurately represented AF. However, the 6 min duration we adopted in this study was based on current guidelines and used as a surrogate for silent AF. In addition, two cardiac electrophysiologists independently reviewed records of AHREs, thereby reducing the error that could result from inadequate judgment.

## 5. Conclusions

A biomarker of inflammation, hs-CRP, could predict the occurrence of subsequent AHREs ≥ 6 min in patients with pacemakers and preserved EF, which contributed to the discrimination of the AHREs risk model. Our results suggest that inflammation could play a role in AF pathogenesis in patients with preserved EF.

## Figures and Tables

**Table 1:** Baseline characteristics of the study participants.

	AHRE	No AHRE	*p* Value
	*N* = 66	*N* = 105	
**Basic characteristics**			
Age (year)	74.8 ± 11.2	73.6 ± 11.8	0.540
Male, no. (%)	36 (54.5)	52 (49.5)	0.315
Body weight, kg	60.8 ± 10.2	62.2 ± 11.4	0.388
Body height, cm	158.7 ± 9.2	159.3 ± 8.6	0.673
Body mass index, kg/m^2^	24.1 ± 3.5	24.5 ± 3.6	0.547
Smoking history, no. (%)	16 (24.2)	19 (18.1)	0.218
Sick sinus syndrome, no. (%)	44 (66.7)	46 (43.8)	0.003
Prior atrial fibrillation ^1^, no. (%)	14 (21.1)	6 (5.7)	0.003
Risk factors for stroke, no. (%)			
History of heart failure	6 (9.1)	14 (13.3)	0.279
Hypertension	45 (68.2)	73 (69.5)	0.492
Diabetes mellitus	19 (28.8)	42 (40)	0.092
Prior stroke/TIA	3 (4.5)	8 (7.6)	0.324
Peripheral artery disease	0	3 (2.9)	0.229
Coronary artery disease	15 (22.7)	21 (20)	0.405
CHADS2Vasc score (0–10)	3.2 ± 1.4	3.4 ± 1.5	0.531
ESRD	2 (3.0)	11 (10.5)	0.063
CKD	6 (9.1)	9 (8.6)	0.557
COPD	5 (7.6)	4 (3.8)	0.232
Medications, no. (%)			
Beta-blocker	10 (15.2)	27 (25.7)	0.073
ACEi/ARB	22 (33.3)	40 (38.1)	0.321
Calcium channel blockers	28 (42.4)	40 (38.1)	0.343
Diuretics	18 (27.3)	31 (29.5)	0.445
Antiarrhythmic drugs	12 (18.2)	12 (11.4)	0.156
White blood cells (cell × 10^3^/mL)	7.5 ± 2.5	7.4 ± 3.1	0.867
Biomarkers			
NT-pro-BNP (pg/mL)	266.7 ± 590.4	168.9 ± 408.9	0.203
hs-CRP (mg/dL)	1.85 ± 2.71	1.19 ± 1.89	0.062
hs-cTnT (ng/L)	42.1 ± 38.5	36.0 ± 41.2	0.332
D-dimer (mg/L)	2.3 ± 2.8	2.1 ± 2.6	0.665
RV pacing (%)	39.3 ± 41.8	39.8 ± 42.3	0.940
**Echocardiographic parameters**			
IVST, mm	11.4 ± 2.2	11.5 ± 2.0	0.835
LVPWT, mm	10.9 ± 2.2	11.0 ± 1.8	0.758
LVEDD, mm	46.7 ± 6.2	46.3 ± 4.8	0.176
LVESD, mm	29.1 ± 5.0	28.3 ± 4.2	0.301
LVEDV, mL	103.1 ± 31.8	100.6 ± 23.4	0.543
LVESV, mL	33.9 ± 14.5	31.4 ± 11.1	0.204
LVEF, %	66.9 ± 7.4	68.2 ± 7.6	0.282
LVM, g	193.8 ± 68.4	192.2 ± 53.7	0.862
LVMI, g/m^2^	120.5 ± 44.2	118.0 ± 33.7	0.687
LA dimension, mm	41.3 ± 7.4	40.7 ± 6.2	0.629
MV E, cm/s	84.0 ± 26.6	78.9 ± 29.3	0.241
MV A, cm/s	81.6 ± 22.3	93.1 ± 31.4	0.006

Values are expressed as mean ± SD or number (percentage); ^1^ except permanent and long-standing persistent AF (Atrial fibrillation). Abbreviation: AHREs, atrial high-rate episodes; TIA, transient ischemic attack; ESRD, end-stage renal disease; CKD, chronic kidney disease; COPD, chronic obstructive pulmonary disease; ACEi/ARB, angiotensin-converting enzyme inhibitors/angiotensin receptor blockers; NT-pro-BNP, N-terminal pro-brain natriuretic peptide; hs-CRP, high-sensitivity C-reactive protein; hs-cTnT, high-sensitivity cardiac troponin T; RV, right ventricle; IVST, interventricular septal thickness; LA, left atrium; LVEDD, left ventricular end-diastolic diameter; LVEDV, left ventricular end-diastolic volume; LVEF, left ventricular ejection fraction; LVESD, left ventricular end-systolic diameter; LVESV, left ventricular end-systolic volume; LVM, left ventricular mass; LVMI, left ventricular mass index; LVPWT, left ventricular posterior wall thickness; MV, mitral valve.

**Table 2:** Predictors of subsequent atrial high-rate episodes in the univariate and multivariate Cox hazards model.

	Univariate			Multivariate ^1^		
	HR	95% CI	*p* Value	HR	95% CI	*p* Value
Age ^2^	1.009	0.988–1.031	0.407	0.998	0.976–1.022	0.889
Gender, male	0.827	0.509–1.344	0.444	0.732	0.445–1.204	0.219
BMI ^2^	0.965	0.902–1.033	0.307			
Smoker	1.228	0.698–2.161	0.475			
SSS	2.074	1.239–3.472	0.005	1.900	1.083–3.333	0.025
Prior AF	3.843	2.078–7.107	<0.001	2.797	1.452–5.388	0.002
CHF	0.752	0.324–1.742	0.505			
Hypertension	1.003	0.597–1.685	0.990			
Diabetes mellitus	0.698	0.409–1.189	0.185			
CVA or TIA	0.699	0.219–228	0.545			
PAD	0.048	0–110.205	0.442			
CAD	1.213	0.681–2.160	0.512			
ESRD	0.335	0.082–1.369	0.128			
CKD	1.236	0.533–2.866	0.621			
COPD	2.001	0.800–5.005	0.138			
Beta–blocker	0.563	0.286–1.111	0.098	0.517	0.256–1.047	0.067
ACEi/ARB	0.869	0.521–1.450	0.591			
CCBs	1.296	0.793–2.119	0.301			
Diuretics	0.964	0.560–1.660	0.894			
AADs	1.697	0.904–3.188	0.100			
White blood cells ^2^	1.014	0.939–1.095	0.716			
LVEF ^2^	0.990	0.959–1.023	0.561			
MV E wave ^2^	1.005	0.997–1.013	0.226			
MV A wave ^2^	0.988	0.979–0.997	0.008	0.989	0.979–0.999	0.038
LA dimension ^2^	1.007	0.971–1.043	0.715			
NT-pro-BNP ^2^	1.000	1.000–1.000	0.072	1.000	1.000–1.001	0.109
Hs-CRP ^2^	1.121	1.024–1.227	0.013	1.121	1.015–1.238	0.024
Hs-TnT ^2^	1.004	0.999–1.009	0.149			
D-dimer ^2^	1.029	0.943–1.123	0.521			

^1^ Adjusted for age, gender, SSS, prior AF, beta-blocker, MV A wave, NT-pro-BNP, hs-CRP. ^2^ For those continuous variables, HR represented the increased risk per unit. Abbreviations: CI, confidence interval; HR, hazard ratio; BMI, body mass index; SSS, sick sinus syndrome; AF, atrial fibrillation; CHF, congestive heart failure; CVA, cerebral vascular attack; TIA, transient ischemic attack; PAD, peripheral artery disease; CAD, coronary artery disease; ESRD, end-stage renal disease; COPD, chronic obstructive pulmonary disease; ACEi/ARB, angiotensin-converting enzyme inhibitors/angiotensin receptor blockers; CCBs, calcium channel blockers; AADs, anti-arrhythmic drugs; LVEF, left ventricular ejection fraction; MV, mitral valve; LA, left atrium; NT-pro-BNP, N-terminal pro-brain natriuretic peptide; hs-CRP, high-sensitivity C-reactive protein; hs-cTnT, high-sensitivity cardiac troponin T.

**Table 3:** Univariate and multivariate linear regression analysis for atrial high-rate episode burden in patients with subsequent atrial high-rate episodes. (*N* = 66).

	Univariate		Multivariate ^1^	
	Standardized Beta	*p* Value	Standardized Beta	*p* Value
Age	0.159	0.202	−0.014	0.901
Gender, male	0.159	0.202	0.048	0.673
BMI	0.025	0.843		
Smoker	−0.096	0.442		
SSS	0.029	0.815		
Prior AF	0.326	0.008	0.279	0.013
CHF	0.402	0.001	0.321	0.004
Hypertension	0.242	0.050	0.241	0.048
Diabetes mellitus	−0.019	0.883		
CVA or TIA	−0.114	0.364		
PAD	–	–		
CAD	−0.047	0.710		
ESRD	−0.096	0.443		
CKD	0.141	0.258		
COPD	0.206	0.098	0.161	0.147
Beta-blocker	0.077	0.537		
ACEi/ARB	0.041	0.743		
CCBs	−0.061	0.625		
Diuretics	0.061	0.629		
AADs	−0.054	0.667		
White blood cells	0.019	0.879		
LVEF	0.096	0.441		
MV E wave	0.066	0.597		
MV A wave	−0.028	0.820		
LA dimension	0.022	0.861		
NT-pro-BNP	−0.041	0.744		
hs-CRP	0.258	0.037	0.223	0.038
hs-TnT	0.039	0.753		
D-dimer	−0.184	0.139		
RV pacing (%)	0.111	0.374		

^1^ Adjusted for age, gender, prior AF, CHF, hypertension, COPD, and hs-CRP. Abbreviations: CI, confidence interval; HR, hazard ratio; BMI, body mass index; SSS, sick sinus syndrome; AF, atrial fibrillation; CHF, congestive heart failure; CVA, cerebral vascular attack; TIA, transient ischemic attack; PAD, peripheral artery disease; CAD, coronary artery disease; ESRD, end-stage renal disease; COPD, chronic obstructive pulmonary disease; ACEi/ARB, angiotensin-converting enzyme inhibitors/angiotensin receptor blockers; CCBs, calcium channel blockers; AADs, anti-arrhythmic drugs; LVEF, left ventricular ejection fraction; MV, mitral valve; LA, left atrium; NT-pro-BNP, N-terminal pro-brain natriuretic peptide; hs-CRP, high-sensitivity C-reactive protein; hs-cTnT, high-sensitivity cardiac troponin T; RV, right ventricle.

## Supplementary Materials

The following are available online at https://www.mdpi.com/2077-0383/9/11/3677/s1: Figure S1: ROC analysis to determine sensitivity and specificity of hs-CRP for AHREs more than 6 min, Table S1: Predictors of Subsequent Atrial High Rate Episodes in Non-prior Atrial Fibrillation Patients by the Univariate and Multivariate Cox Hazards Model (*N* = 151), Table S2: Predictors of Subsequent Atrial High Rate Episodes in by the Univariate and Multivariate Subdistribution Hazard Model.
