# Supplementary material for: High-Sensitivity C-Reactive Protein is a Predictor of Subsequent Atrial High-Rate Episodes in Patients with Pacemakers and Preserved Ejection Fraction
Source: J Clin Med. 2020 Nov 16;9(11):3677. doi: 10.3390/jcm9113677 (PMC7696571; doi:10.3390/jcm9113677)
Supplement: Supplementary file 1 [file jcm-09-03677-s001.pdf]

Supplement Table S1. Predictors of Subsequent Atrial High Rate Episodes in Non-prior Atrial Fibrillation Patients by the Univariate and Multivariate Cox Hazards Model (N=151).

	**Univariate**			**Multivariate**[1]		
	**HR**	**95% CI**	***P value***	HR	95% CI	*P value*
Age[2]	1.009	0.986-1.034	0.441	1.001	0.977-1.025	0.958
Gender, male	0.687	0.349-1.197	0.185	0.636	0.360-1.124	0.119
BMI[2]	0.951	0.879-1.029	0.209			
Smoker	1.344	0.726-2.488	0.346			
SSS	1.775	1.011-3.118	0.046	2.050	1.129-3.722	0.018
CHF	0.667	0.240-1.854	0.438			
Hypertension	1.046	0.580-1.886	0.881			
Diabetes mellitus	0.815	0.456-1.455	0.489			
CVA or TIA	0.591	0.144-2.430	0.465			
PAD	0.048	0-543.985	0.542			
CAD	1.540	0.833-2.847	0.169			
ESRD	0.199	0.027-1.440	0.110			
CKD	1.279	0.508-3.222	0.602			
COPD	1.950	0.604-6.296	0.264			
Beta-blocker	0.498	0.222-1.114	0.090	0.600	0.267-1.348	0.216
ACEi/ARB	1.060	0.606-1.853	0.839			
CCBs	1.406	0.809-2.442	0.227			
Diuretics	1.083	0.593-1.978	0.795			
AADs	1.529	0.686-3.405	0.299			
White blood cells[2]	1.040	0.963-1.124	0.317			
LVEF[2]	0.981	0.945-1.018	0.317			
MV E wave[2]	1.006	0.997-1.014	0.213			
MV A wave[2]	0.987	0.977-0.998	0.015	0.987	0.977-0.998	0.018
LA dimension[2]	1.005	0.966-1.046	0.798			
NT-pro-BNP[2]	1.000	1.000-1.001	0.167			
hs-CRP[2]	1.134	1.026-1.253	0.014	1.155	1.038-1.285	0.008
hs-TnT[2]	1.004	0.998-1.010	0.153			
D-dimer[2]	1.036	0.941-1.142	0.470			

[1] Adjusted for age, gender, SSS, beta-blocker, MV A wave, hs-CRP. [2] For those continuous variables, HR represented the increased risk per unit. Abbreviations: CI, confidence interval; HR, hazard ratio; BMI, body mass index; SSS, sick sinus syndrome; CHF, congestive heart failure; CVA, cerebral vascular attack; TIA, transient ischemic attack; PAD, peripheral artery disease; CAD, coronary artery disease; ESRD, end-stage renal disease; COPD, chronic obstructive pulmonary disease; ACEi/ARB, angiotensin-converting enzyme inhibitors/angiotensin receptor blockers; CCBs, calcium channel blockers; AADs, anti-arrhythmic drugs; LVEF, left ventricular ejection fraction; MV, mitral valve; LA, left atrium; NT-pro-BNP, N-terminal pro-brain natriuretic peptide; hs-CRP, high-sensitive C-reactive protein; hs-cTnT, high-sensitive cardiac troponin T.

Supplement Table S2. Predictors of Subsequent Atrial High Rate Episodes in by the Univariate and Multivariate Subdistribution Hazard Model

	Univariate			Multivariate[1]		
	**HR**	**95% CI**	***P value***	HR	95% CI	*P value*
Age[2]	1.010	0.989-1.031	0.377	0.999	0.977-1.023	0.965
Gender, male	0.811	0.500-1.317	0.398	0.716	0.436-1.174	0.185
BMI[2]	0.967	0.905-1.034	0.325			
Smoker	1.200	0.683-2.109	0.526			
SSS	2.245	1.342-3.754	0.002	2.292	1.318-3.987	0.003
Prior AF	3.888	2.102-7.191	<0.001	2.569	1.342-4.918	0.004
CHF	0.684	0.295-1.586	0.376			
Hypertension	0.958	0.570-1.610	0.872			
Diabetes mellitus	0.672	0.394-1.146	0.144			
CVA or TIA	0.677	0.212-2.157	0.509			
PAD	0.047	0-60.623	0.404			
CAD	1.242	0.698-2.211	0.462			
ESRD	0.329	0.080-1.344	0.122			
CKD	1.266	0.546-2.936	0.583			
COPD	1.607	0.644-4.009	0.309			
Beta-blocker	0.568	0.289-1.116	0.101			
ACEi/ARB	0.849	0.509-1.416	0.529			
CCBs	1.254	0.769-2.044	0.364			
Diuretics	0.929	0.540-1.596	0.789			
AADs	1.607	0.858-3.011	0.139			
White blood cells[2]	1.004	0.930-1.085	0.918			
LVEF[2]	0.989	0.958-1.021	0.513			
MV E wave[2]	1.005	0.997-1.013	0.244			
MV A wave[2]	0.988	0.979-0.997	0.009	0.990	0.980-1.000	0.990
LA dimension[2]	1.005	0.970-1.042	0.784			
NT-pro-BNP[2]	1.000	1.000-1.001	0.071	1.000	1.000-1.001	0.106
hs-CRP[2]	1.121	1.023-1.227	0.014	1.122	1.014-1.242	0.026
hs-TnT[2]	1.004	0.998-1.009	0.164			
D-dimer[2]	1.023	0.935-1.118	0.623			

[1] Adjusted for age, gender, SSS, prior AF, MV A wave, NT-pro-BNP, hs-CRP. [2] For those continuous variables, HR represented the increased risk per unit. Abbreviations: CI, confidence interval; HR, hazard ratio; BMI, body mass index; SSS, sick sinus syndrome, AF, atrial fibrillation; CHF, congestive heart failure; CVA, cerebral vascular attack; TIA, transient ischemic attack; PAD, peripheral artery disease; CAD, coronary artery disease; ESRD, end-stage renal disease; COPD, chronic obstructive pulmonary disease; ACEi/ARB, angiotensin-converting enzyme inhibitors/angiotensin receptor blockers; CCBs, calcium channel blockers; AADs, anti-arrhythmic drugs; LVEF, left ventricular ejection fraction; MV, mitral valve; LA, left atrium; NT-pro-BNP, N-terminal pro-brain natriuretic peptide; hs-CRP, high-sensitive C-reactive protein; hs-cTnT, high-sensitive cardiac troponin T.

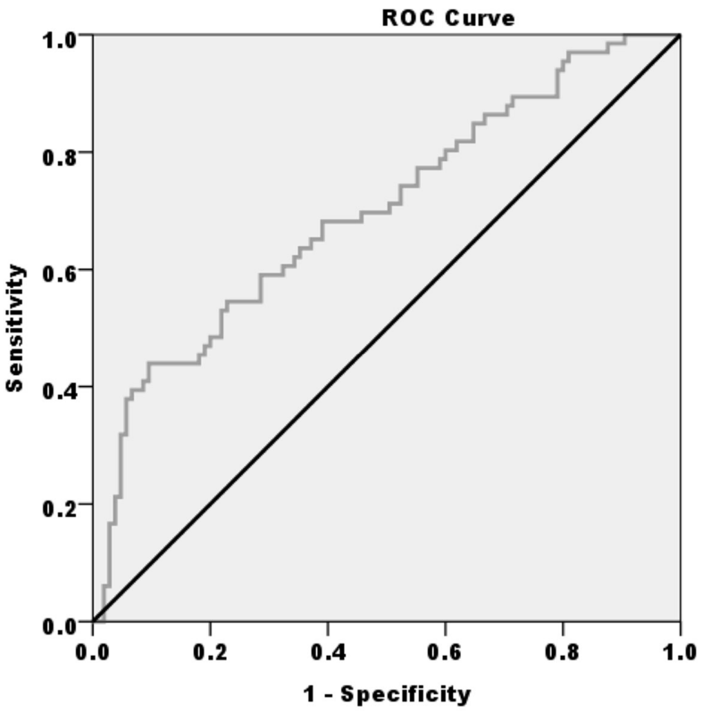

Supplement Figure S1. ROC analysis to determine sensitivity and specificity of hs-CRP for AHREs more than 6 minutes. The optimized cut-point value of hs-CRP was 0.525 mg/L. ROC, receiver operating characteristic; hs-CRP, high-sensitivity C-reactive protein
